# Letter to the editor regarding “Kara M, Alp G, Pekdiker M, Ketenci S, Porreca A et al. Evaluating leflunomide and methotrexate combination vs. monotherapy in rheumatoid and psoriatic arthritis. Turkish Journal of Medical Sciences: 2025; 55 (3): 632–643”

**DOI:** 10.55730/1300-0144.6090

**Published:** 2025-10-07

**Authors:** Özge TEZEN

**Affiliations:** Department of Physical Medicine and Rehabilitation, Ankara Bilkent City Hospital, Ankara, Turkiye

I read with great interest the article by Kara et al. titled “Evaluating leflunomide and methotrexate combination vs. monotherapy in rheumatoid and psoriatic arthritis” [1]. The study provides valuable clinical data, particularly in settings where access to biologic therapies is limited. However, several methodological and clinical limitations warrant further discussion.

First, the relatively small sample size, particularly in the psoriatic arthritis subgroup, restricts the statistical power and generalizability of the findings. Larger multicenter studies are therefore necessary [2]. Second, although elevations in liver enzymes were reported, data on long-term hepatic risks (such as fibrosis indices), survival outcomes, and reasons for treatment discontinuation were not provided. The short follow-up period may have been insufficient to capture long-term safety outcomes, including hepatotoxicity and hematologic toxicities, which are well-documented with both methotrexate (MTX) and leflunomide (LEF) [3]. Furthermore, radiographic progression and comprehensive patient-reported outcomes (including functional status, fatigue, and work capacity) are now standard components of in rheumatology research, and their omission limits the clinical applicability of the results [4,5]. Finally, given the known teratogenic effects of both MTX and LEF, the lack of reproductive counseling and vaccination strategies constitutes a practical gap for clinicians [6].

In conclusion, while the article offers important comparative data, future research with larger cohorts, extended follow-up periods, and comprehensive patient reported outcomes is encouraged to better define the optimal treatment strategy. Additionally, incorporation of radiographic assessments, patient-reported outcomes, and reproductive health considerations would further enhance the clinical relevance.
